# Image-guided stereotactic body radiation therapy (SBRT): an emerging treatment paradigm with a new promise in radiation oncology

**DOI:** 10.2349/biij.3.1.e5

**Published:** 2007-01-01

**Authors:** BS Teh

**Affiliations:** Department of Radiology, Baylor College of Medicine and Radiation Therapy Department, The Methodist Hospital; Texas, United States

Stereotactic radiosurgery (SRS), coupling radiation with a stereotactic guiding device, was first performed by Leksell in 1951 [1]. SRS is now an established treatment option for many benign and malignant tumours. With the advances in technology, including patient/target immobilisation, tumour/target tracking, image-guidance, and radiation planning and delivery, SRS to extracranial sites has become a reality. Extracranial stereotactic radiosurgery/radioablation or stereotactic body radiation therapy (SBRT) is defined by the American Society for Therapeutic Radiology and Oncology (ASTRO) and the American College of Radiology (ACR) as a “treatment method to deliver a high dose of radiation to the target, utilising either a single dose or a small number of fractions with a high degree of precision within the body [2].”

Like any novel therapeutic approach in medicine, SBRT needs to be performed with caution and ideally in the clinical trial setting, especially in view of the biologically potent dose prescription as high as 20 to 30 Gy per fraction. Practice guidelines should be followed to avoid the risk of severe complications [2]. Teamwork is essential for the success of this new treatment, and the team should include not only medical physicists, but also dosimetrists, radiation therapists, nurses, radiologists, and radiation oncologists. Some essential components needed for the clinical implementation of SBRT include: patient immobilisation and accurate reposition from simulation session to each treatment session, accounting for motion or tracking “moving target” e.g., lung tumours, fusion of various imaging studies, construction of tight dose distributions covering tumour with rapid fall-off the adjacent normal tissues, as well as the availability of image-guidance. The article in this issue of Biomedical Imaging and Intervention Journal [3] illustrates the proper conduct of SBRT, which includes the use of immobilisation device, accurate repositioning of the patient with KV-X Ray as image-guidance, 4D-CT to account for tumour motion, proper fusion of PET/CT and MRI with simulation CT, use of visicoil/bony landmarks for image-guidance, and construction of tight isodose around the tumour.

Radiobiologically, the dose fraction regimens used in SBRT ranging from 6 to 30Gy, are aimed to yield substantially more potent biological and clinical effects. Applying linear-quadratic formula, Fowler and colleagues have compared the relative biological effectiveness of various SBRT fractionation schemes with the conventional fractionation scheme for non-small cell lung cancer [4]. The conventional 60Gy in 30 fractions (2 Gy per fraction) and 60Gy in 3 fractions (20Gy per fraction) have biological equivalent doses (BED) of 72Gy and 180Gy, respectively, as well as can yield an estimated progression free survival at 30 months of 15% and >99% respectively. Therefore, SBRT approach is especially beneficial for treating more radio-resistant tumours, such as, non-small cell lung cancer, melanoma, and renal cell carcinoma as illustrated by Teh *et al* [3], both in primary and metastatic settings.

Clinical experience is most vast in the three extracranial sites, namely lung, liver, and spine. Small non-small cell lung cancers (Stage I and II) in either operable or medically inoperable patients as well as metastatic lung lesions have been treated with SBRT. Doses as high as 60Gy in 3 fractions were used without any significant complication [4-6]. A multi-institutional retrospective study from Japan showed that patients treated with SBRT have a similar survival when compared to patients treated surgically, but with less treatment-related morbidity [7]. Radiation Therapy Oncology Group (RTOG) is currently running a Phase II trial addressing SBRT in early non-small cell lung cancer in medically inoperable patients. Similar promising local control and favourable toxicity profiles were achieved using SBRT in primary hepato-cellular carcinoma (HCC) and metastatic liver lesions [8-10]. SBRT or SRS to the spine enables re-treatment after the initial conventional fractionated radiotherapy. Special attention needs to be paid to the spinal cord, especially in previously irradiated patients. Again, the local control rates, including pain relief with SBRT, have been very satisfactory with minimal side-effects [11-13]. Similar results were reported by Teh *et al* in this issue of ***biij*** [3].

SBRT is an emerging treatment paradigm with a new promise in radiation oncology. The promise to produce biologically potent dose in a shorter period of time and a non-invasive manner is very attractive. SBRT can also be applied to patients with metastatic disease for cyto-reduction in combination with chemotherapy and for more durable and faster symptoms palliation. Nevertheless, many aspects of SBRT need further investigations and research. What is the best SBRT fractionation scheme? Is the best scheme dependent on cancer type, organ site, tumour size, degree of hypoxia, etc? Many other radiobiological questions also need answers, e.g., the optimal radiobiological model for tumour and normal tissues when treated with SBRT and the mechanism of SBRT in overcoming radio-resistance. Clinically, besides patient’s symptoms evaluation, there is still debate on the best imaging modality for follow-up as one can still see the stable tumour mass on CT for some time post-treatment. Functional biological imaging like PET/CT may be better than the conventional anatomic imaging. Future work should also look into the effects of SBRT in combination with novel targeted agents, such as, EGFR-inhibitor and VEGF-inhibitor.

## ACKNOWLEDGEMENT

The author is a recipient of The Methodist Hospital Research Institute research grant.
